# Fracture Behavior and Digital Image Analysis of GFRP Reinforced Concrete Notched Beams

**DOI:** 10.3390/ma15175981

**Published:** 2022-08-30

**Authors:** Mohammod Minhajur Rahman, Xudong Zhao, Tommaso D’Antino, Francesco Focacci, Christian Carloni

**Affiliations:** 1 Department of Civil and Environmental Engineering, Case Western Reserve University, Cleveland, OH 44106, USA; 2 Department of Architecture, Built Environment, and Construction Engineering (ABC), Politecnico di Milano, 20133 Milan, Italy; 3 eCampus University, 22060 Novedrate, Italy

**Keywords:** notched beam, GFRP bar, three-point bending test, digital image correlation

## Abstract

This study presents three-point bending fracture tests on glass fiber-reinforced polymer (GFRP) reinforced concrete notched beams. Few studies have been conducted to date to understand the fracture behavior of this type of specimens. The specimens have nominal depth, width, and length equal to 150 mm, 150 mm, and 550 mm. Plain concrete notched beams with the same dimensions are cast from the same batch of concrete to compare the responses with GFRP reinforced concrete notched beams. The notch of the plain concrete specimens is either saw cut or cast. These two notch fabrication methods are compared based on the load responses. The peak load, crack mouth opening displacement (CMOD), GFRP bar slip at two ends, and load point displacement are used to discuss the results of the fracture tests. In addition, digital image analysis is performed to identify the fracture process zone (FPZ) and the location of the neutral axis, which are used to determine the force in the GFRP bar via cross-sectional analysis. Finally, the GFRP bar force versus slip responses are compared with those from the pull-out tests performed on the same bar to show that the bond of the bar in the pull-out tests represents an upper bound limit compared to the behavior in bending.

## 1. Introduction

Glass fiber-reinforced polymer (GFRP) bars have become a major alternative to traditional reinforcing steel bars in concrete structures, and they have been perceived by many experts as a promising material in reinforced concrete (RC) technology [1]. GFRP bars are made of continuous glass fibers embedded in a polymeric resin matrix, often with the addition of additives and fillers [2]. The surface of the bar can be treated with coarse sand and carbon fibers to improve the bond with the concrete, or it can be shaped with ribs similarly to deformed steel bars.

There are several advantages in using GFRP bars as reinforcement in concrete members instead of traditional steel bars: (1) GFRP bars are lightweight and corrosion free compared to steel bars; (2) they have high tensile strength and high stiffness-to-weight ratio; (3) they are more durable than steel bars; (4) they are transparent to electromagnetic fields; and (5) RC structures with GFRP bars can be easily demolished if necessary [3,4]. Depending upon the geographical location, GFRP bars can also be cost effective compared to the traditional choices of reinforcement in concrete members [5].

In recent times, GFRP reinforced concrete member has been used extensively in many civil engineering projects. In sensitive projects, such as seawalls, dams, and power plants, designers are prone to use GFRP bars due to their corrosion resistance. Piles reinforced with GFRP bars are found to be more durable due to resistance to corrosion compared to piles reinforced with steel bars [6]. For similar reasons, GFRP bars can also be used in bridge deck slabs as an alternative to steel expansion joints [7] where other types of FRP (i.e., carbon [8] and basalt [9] FRP bars) are used as well. GFRP bars are also used in transportation infrastructure where magnetic fields would increase the steel corrosion rate, airport runways [10], and mining and tunneling where the tunnel boring machine can easily cut the FRP bars [11].

In the past, researchers studied extensively FRP reinforced concrete members and established the design guidelines [12,13,14]. However, very limited research has been conducted to correlate the fracture behavior (i.e., the crack opening) of concrete to the bond of the GFRP bars [7,8,9,15,16]. Typically, the elastic modulus of GFRP bars is 20–25% of the elastic modulus of steel. Due to the low stiffness of GFRP reinforced concrete members compared to steel reinforced concrete members, the design process of the former largely depends on the opening of the flexural and shear-flexural cracks and deformability of the members [17]. Moreover, for most of the structural members, the crack propagation is the prime cause of structure deterioration. Thus, using fracture mechanics to analyze GFRP reinforced concrete members seems appropriate. The three-point bending (TPB) fracture test of GFRP reinforced notched beams can be used to investigate the crack pattern and the relationship between the crack opening and the bond between the bar and concrete. To this end, the displacement field obtained using digital image correlation (DIC) on the lateral face of the beam allows the determination of the strain field and crack opening to have an in-depth analysis of the notched beam specimen.

Few studies were conducted to examine the fracture behavior of steel-reinforced concrete beams using a TPB test set-up. The location of the steel bar in the concrete beam was varied in [18], and it was found out that the peak load increased when the steel bar was placed through the notch compared to when it was not placed through the notch, while the crack mouth opening displacement (CMOD) [19] showed an opposite trend. Other studies examined fracture tests of steel-reinforced concrete beams by selecting different crack to depth ratios [20] and different depths of the beam [21]. In [20], it was found that the fracture toughness is independent of the crack to depth ratio when it is equal to or greater than 0.4. In [21], the height of the specimen was found not to have any effect on the fracture toughness and could be regarded as a constant. Recently, Chai et al. [7] conducted a test campaign to examine the fracture behavior of basalt FRP reinforced concrete notched beams by varying the cover thickness and the diameter of the bars. They found that as the bar diameter increases, the crack kinking angle, peak load, and load point displacement increase as well. The study also found that specimens with smaller cover thickness give higher peak load and deflection (with some exceptions). However, similar studies are not available for GFRP reinforced concrete beams. It is important to extend the research to other types of FRP bar, as the market variability introduces different surface conditions for these bars, which results in unique mechanical and physical properties at the interface with concrete.

In this study, the fracture behavior of GFRP reinforced concrete notched beams is examined through TPB tests. Fracture test parameters, namely the CMOD, load point displacement, and peak load, are obtained and analyzed. Plain concrete notched beams are also tested and their behavior compared with the GFRP reinforced notched beams. The effect of casting the notch rather than saw cutting it after the concrete is cured is also investigated by testing plain concrete beams with both cast initial notch and saw cut notch. Finally, DIC is used to obtain the displacement and strain fields on the beam lateral face. The load point displacement (LPD) is also obtained from DIC analysis and compared with the LPD obtained from linear variable displacement transformers (LVDTs). Strain and displacement fields and contour plots from DIC analysis are presented to study the characteristics of the fracture process zone (FPZ) [22,23,24,25]. Finally, the GFRP bar force and slip are computed using cross-sectional analysis of the notched beam and DIC. The results of TPB tests are compared with those of the pull-out tests [5] of the same GFRP bars embedded in concrete with the overarching goal of identifying any difference in the bond mechanism of these two tests.

## 2. Research Significance

This study is conducted to gather in-depth knowledge of the fracture behavior of GFRP reinforced notched beams. The bond between the bar and concrete is a key aspect to investigate the use of GFRP bars in reinforced concrete. This paper tackles two aspects: (1) the relationship between the pull-out tests and bending tests; (2) the relationship between the opening of a flexural crack and the slip of the bar.

## 3. Materials and Testing Methods

In this Section, the experimental campaign is described. First, the mechanical properties of the concrete and the reinforcing GFRP bar are presented. Then, the geometrical dimensions and the preparation of the specimens for the TPB tests are described. Finally, the test set-up and the procedure are explained.

### 3.1. GFRP Bars and Concrete

All the notched beam specimens and the cylinders for material characterization were cast from the same batch of concrete. The mixture proportions of different constituents of the concrete by weight were cement (2.6):water (1):fine aggregates (10.3):coarse aggregates (10.5). The maximum aggregate size was 12 mm. The slump test was performed at the beginning of the casting process according to [26], and the value of the slump was 152 mm. All the beams and cylinders were covered with plastic sheets after the casting process was completed. After 24 h, the specimens were demolded and covered with wet burlap and plastic sheets. The specimens were left in the same curing condition until they were tested. The test campaign started after 33 days from the casting day.

**Figure 1:**
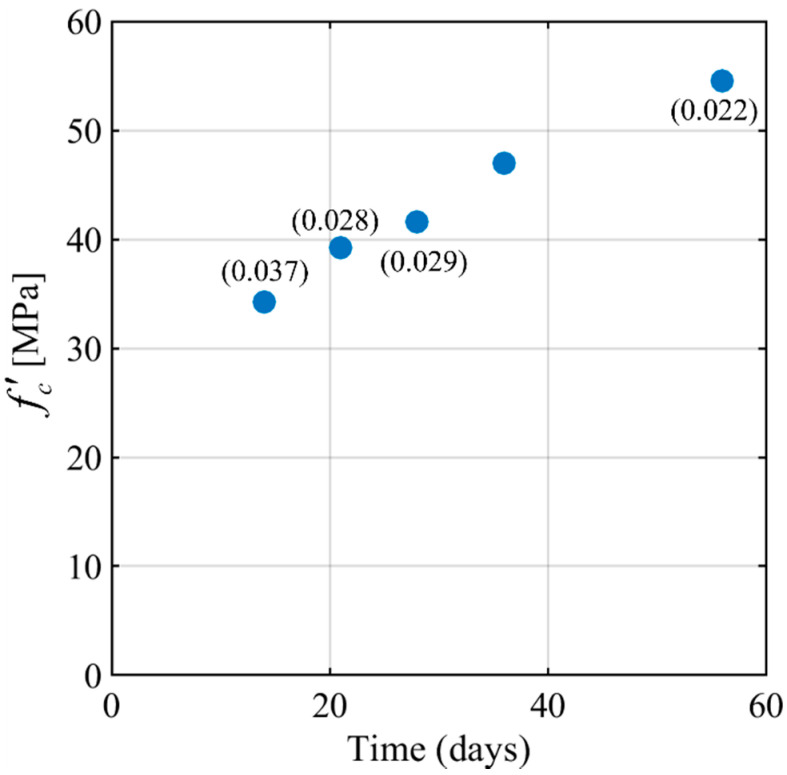
Average concrete cylinder compressive strength (${f^{\prime }}_{c}$) versus time.

Compressive tests were performed on 150 mm (diameter) by 300 mm (height) cylinders at different ages (14, 21, 28, 36, and 56 days) according to [27]. At 28 days, the average compressive strength, ${f^{\prime }}_{c}$, of three concrete cylinders was 41.6 MPa, and the coefficient of variation (CoV) was 0.029. In Figure 1, the average compressive strength (from testing three cylinders) is plotted against the age of concrete (in days counted from the day of casting). The corresponding CoV is reported within parentheses.

The tensile strength of concrete was obtained using the following formula from ACI 318-11 [28]:

(1) $${f^{\prime }}_{t}=k{\left({f^{\prime }}_{c}\right)}^{n}$$

where the average compressive strength at 56 days was considered. The values of *k* and *n* were 0.56 and 0.5, respectively, according to ACI 318-11 [28]. The tensile strength, ${f^{\prime }}_{c}$, was found to be 4.1 MPa.

**Figure 2:**
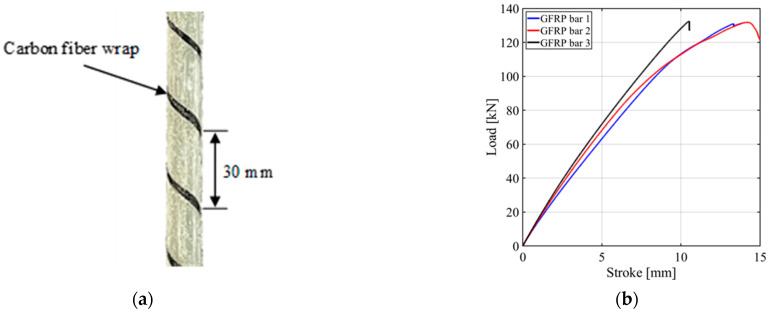
Materials for the test campaign: (**a**) Photo of a segment of the GFRP bar showing the spirally wrapped carbon yarn; (**b**) Tensile test results of GFRP bars.

Four notched beams were reinforced with a GFRP bar. The GFRP bars were sand coated and were wrapped spirally with a carbon yarn (Figure 2a). According to the manufacturer’s datasheet [29], the calculated bar diameter *d_b_* was 12.7 mm, and the calculated cross-sectional area (*A_bar_*) was 127 mm^2^. The tensile modulus of elasticity of the bar (*E_bar_*) was 46 GPa [29], and the ultimate average tensile load was 120 kN. The tensile strength of the bar was 950 MPa as per the manufacturer’s datasheet.

Three bars were tested to determine the average tensile strength and the load versus stroke curves; the results of these tests are shown in Figure 2b. The ultimate load, tensile strength, and average tensile strength along with its CoV for the three tests are provided in Table 1. Additional details on the tensile tests are reported in [5].

### 3.2. Specimen Preparation and Test Methods

**Figure 3:**
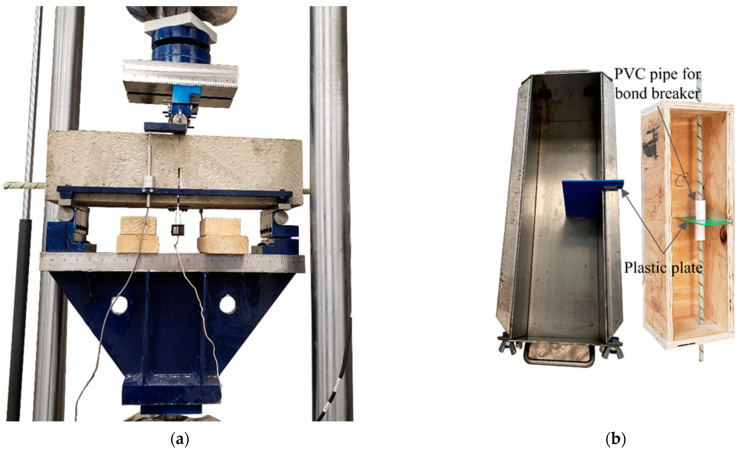

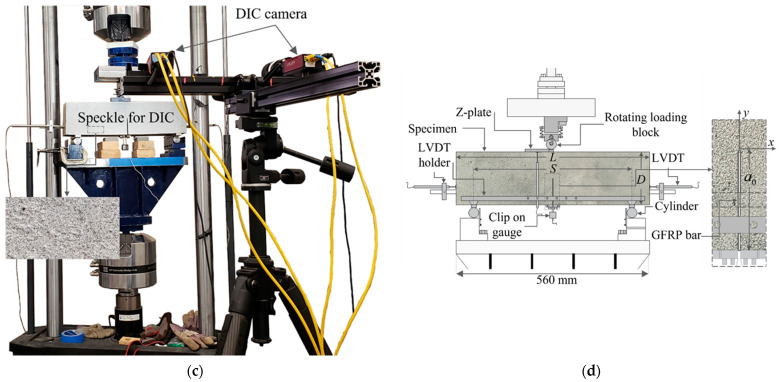
Test set-ups and molds: (**a**) Photo of the test set-up; (**b**) Molds for the GFRP bar reinforced beam and cast notch beam; (**c**) DIC set-up for a test; (**d**) Configuration of the test set-up.

In total, nine notched beams were tested in a three-point bending test configuration. Four out of nine beams were reinforced with a GFRP bar. A photo of the test set-up and the specimen is shown in Figure 3a. The nominal depth (*D*), width (*B*), and length (*L*) of the specimens were 150 mm, 150 mm, and 550 mm, respectively. The span (*S*) was three times the depth of the specimens. A 75 mm long and 3 mm wide concrete notch (*a*_0_) was either cast or saw cut at the midspan cross-section for all specimens.

The GFRP bar extended beyond the concrete specimens at both ends. This extension was necessary to allow for the measurement of the bar slip at the free ends of the bar. A photo of the wooden mold used for these specimens is shown in Figure 3b. A notch with a V-shaped tip was cast for these four specimens using a 3D printed plastic plate. The plastic plate was designed in a way to facilitate the removal process after concrete casting. It should be mentioned that after the beams were cast, the plastic plates were removed after seven days to allow the notch area to gain enough strength to sustain the removal process of the plastic plates and avoid any micro-damage near the tip of the notch.

The plastic plate had a hole of 23 mm diameter. A 50 mm long PVC pipe was used on each side of the plastic plate to create a bond breaker. The PVC was bonded to the plastic plate with silicon, which was also used to seal the gap between the bar and the PVC pipe at the other end of the pipe itself. The bond breaker was necessary to avoid any cone failure around the notch surface during the test. The PVC pipe created a bond breaker of total length, *l* = 100 mm, centered with respect to the notch. The internal diameter of the PVC pipe was 20 mm. The concrete was poured from the side face of the notched beams, i.e., the notch front was perpendicular to the troweled face of the specimen. This orientation allowed having the molded faces as the top and bottom faces of the specimen when it was placed in the testing rig. Three more specimens were cast in a similar manner (i.e., with a cast notch) as described above, except they were cast without the bar (the steel mold used to cast these specimens is shown in Figure 3b). Lastly, two specimens were cast without any initial notch or the bar. For these two specimens, a notch was saw cut with a V-shaped tip diamond blade with water cooling before the tests were performed.

One of the two longitudinal side faces of five notched beams was painted with non-reflective white paint, and then, a black dot pattern was obtained by using a paint spray to prepare the surface of the specimens for the digital image correlation (DIC). The DIC set-up is shown in Figure 3c.

Prior to testing each notched beam (with or without the bar), three measurements were taken for each of the dimensions, i.e., depth (*D*), width (*B*), and length (*L*). In addition, after the test was completed, three measurements of the length of the initial notch (*a*_0_) were taken (for the specimens with the GFRP bar, it was not possible to take the measurements of the initial notch after the test was completed, as the specimens were not fully broken into two halves). The average of these measurements along with their CoV are presented in Table 2. A schematic of the test set-up is shown in Figure 3d. The three-point bending set-up had two steel cylinders placed apart as the loading span (*S*) of the specimens. These two cylinders worked as supports for the specimen and rolled on the steel blocks attached to the base. Another cylinder was placed on top of the specimen to apply the load at the midspan. Springs were used to hang the cylinder from a spherically seated block that was attached to the top head of the testing machine. Two steel bearing plates were glued to the bottom of the specimen and were placed on top of the supporting cylinders to reduce the friction between the concrete and the rotating support cylinders. The cylinders had a radius of 0.2*D*, the steel bearing plates had a width of 0.17*D*, and the depth of the steel bearing plate was half of its width. The steel fixtures were cleaned of any dust and debris before each test. On the top face of the specimen, between the loading cylinder and concrete, a Z-shaped steel plate was used. This Z-shaped plate had a V-shaped section on the top face, so that the loading cylinder rested firmly on it. Both the Z-shaped plate and the steel bearing plates were longer than the width (*B*) of the specimen. Two steel bars with semi-spherical- and cylindrical-shaped supports were placed on the extended part of the steel bearing plates on both sides of the specimen. Each steel bar was equipped with a linear variable displacement transformer (LVDT) that reacted off the flange of the Z-shaped plate placed on top of the specimen. The average of these two LVDT readings is named the load point displacement (Δ). It should be mentioned that when the DIC was used, only one LVDT was used to measure the load point displacement. For some specimens with GFRP bars, two additional LVDTs were used to measure the free end slip of the bar at the end cross-sections of the concrete beam. Two aluminum rings were glued to the protruding portion of the bars at the two ends of the concrete beams. The rings were used to mount two aluminum plates that held the LVDTs, which reacted against the surface of concrete. Prior to testing, two C-shaped steel plates were glued on the bottom surface of the notched beams near the edges of the initial notch. Two knives were then screwed in on the C-shaped plates. The knives were used to mount the clip-on-gauge. The TPB test set-up used in this study was based on the draft of the ACI/ASCE 446 Technical Committee report, which was used in [30,31]. The clip-on-gauge was used to measure the crack mouth opening displacement (CMOD) and to control the test. The rate of the CMOD was chosen to reach the peak load of the specimen within 150 s to 210 s from the beginning of the test. For the specimens without the GFRP bars, the test was started with a CMOD rate of 0.0002 mm/s. At the beginning of the softening branch of the response and specifically at 85% of the peak load, the CMOD rate was increased to 0.0005 mm/s. The rate was increased to 0.001 mm/s when 35% of the peak load was reached in the descending (softening) branch of the response. For those specimens with the GFRP bar, the CMOD rate was the same as the other specimens until the peak load. After the start of the softening region, the CMOD rate was increased 3, 5, 10, and 20 times at different points of the tests.

The specimens were named as follows. Specimens reinforced with a GFRP bar were named as PN_B_X. Notched beam specimens without a GFRP bar for which the notch was cast were named as PN_X. Finally, specimens without a GFRP bar for which the notch was saw cut prior to testing were named as CN_X. X is the number of the specimen.

## 4. Experimental Results

In this Section, the experimental results of the nine TPB tests on notched beams with and without a GFRP bar reinforcement are reported. Table 2 reports the peak load (*F_max_*) of each test and the average along with its CoV for each group (i.e., PN_B_X, PN_X, and CN_X). For the PN_B_X specimens, the first peak load before the descending part was considered as *F_max_*.

### 4.1. Load Responses

**Figure 4:**
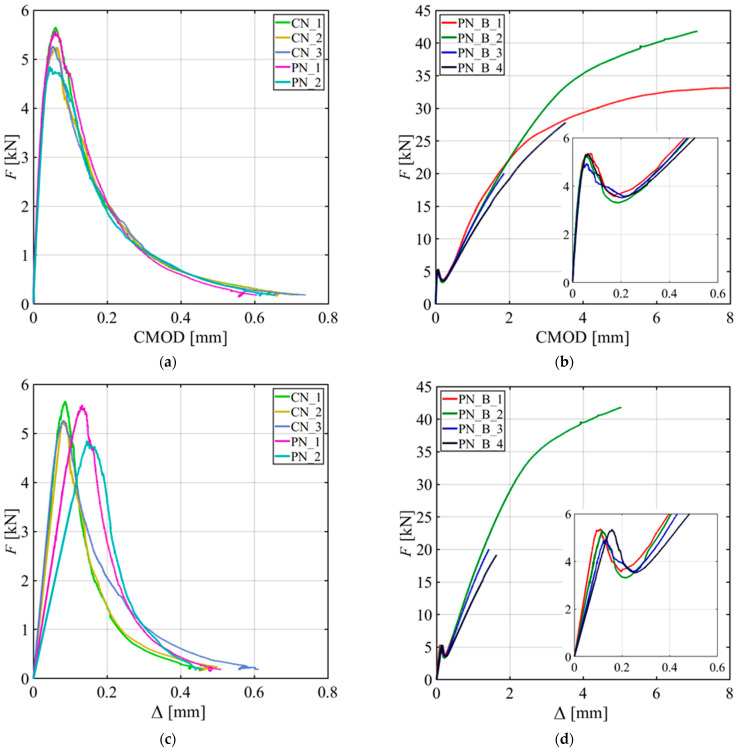
Load responses: (**a**) *F*-CMOD responses for CN_X and PN_X series specimens; (**b**) *F*-CMOD responses for PN_B_X series specimens with a call out for the first segment of the tests; (**c**) *F*-Δ responses for CN_X and PN_X series specimens; and (**d**) *F*-Δ responses for PN_B_X series specimens with a call out for the first segment of the tests.

Figure 4a shows the applied load (*F*) versus the crack mouth opening displacement (CMOD) responses of all the specimens without a GFRP bar reinforcement. Figure 4b shows the *F*-CMOD responses of the four notched beams with a GFRP bar reinforcement. A call out of the initial portion of the response is also provided in Figure 4b. All the specimens show a linear part followed by a nonlinear response before reaching the peak load (*F_max_*).

The post-peak behavior of the CN_X and PN_X specimens features a descending portion characterized by a long tail until the test was stopped at almost zero load. The responses for CN_X and PN_X specimens are consistent with each other, which indicates that casting rather than sawing the notch had no effect. For the PN_B_X specimens, the post-peak behavior shows a descending part followed by an ascending part that starts, on average, at 66% of *F_max_*. It can be assumed that in the post-peak ascending part of the test, the variation of the force in the GFRP bar with respect to the CMOD is higher when compared to the same variation in the preceding part of the response. For PN_B_1 and PN_B_2, the tests were stopped due to safety reasons when the supporting cylinders reached the outer edge of the steel bearing plates. PN_B_3 and PN_B_4 tests were stopped even earlier due to some technical issues.

Specimen PN_B_1 was also stopped earlier (CMOD ~ 2.2 mm) due to the same technical issues; however, it was retested further in stroke control. Figure 4c,d show the applied load (*F*) versus the load point displacement (Δ) response for the CN_X and PN_X specimens and PN_B_X specimens, respectively.

**Figure 5:**
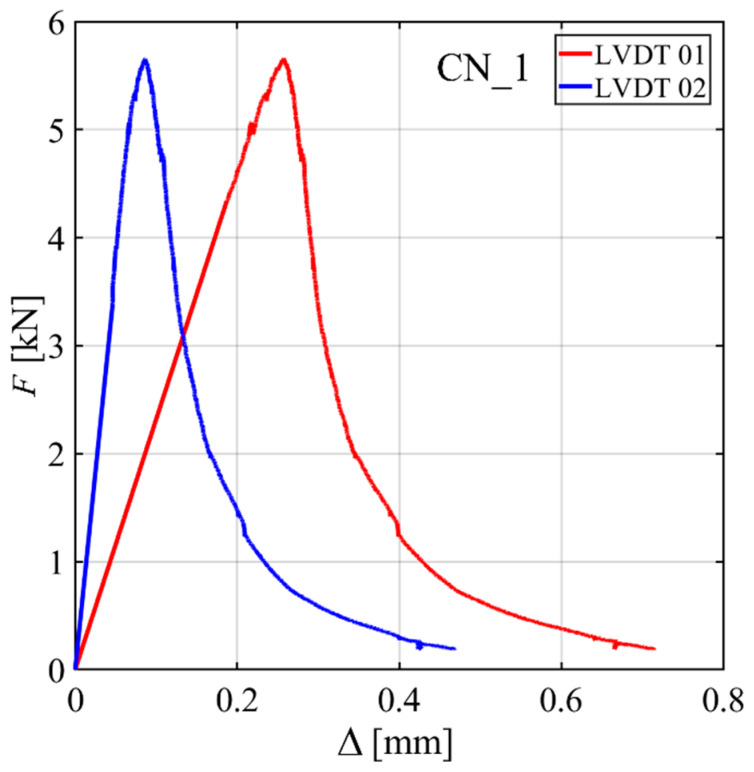
Inconsistency between LVDT readings to measure Δ for CN_1.

The *F*-Δ responses of different groups of specimens show slightly different initial slopes from each other. This inconsistency in the *F*-Δ responses might be originating partly from small adjustments of the specimens and from rotation of the LVDT holders during the test. This kind of trend was observed in previous works [32]. For some specimens, it was also observed that when one LVDT was used on each side of the specimen (CN_1, CN_3, and PN_1) to measure Δ, the two LVDT readings on the two sides were not always consistent with each other. Figure 5 shows this inconsistency for CN_1.

It should be mentioned that during the PN_B_1 retest, the LVDTs that measured Δ were not used due to some technical issues. 

**Figure 6:**
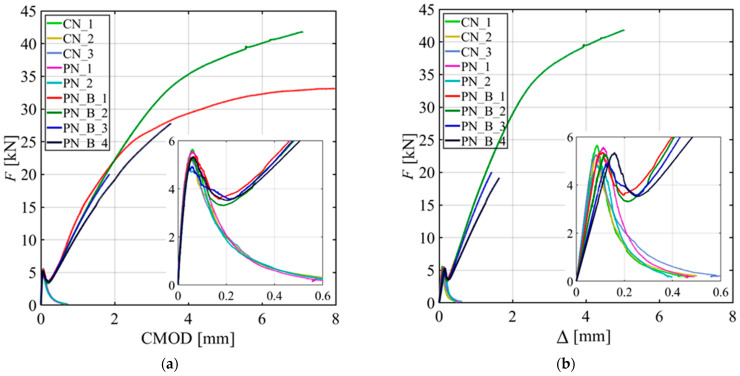
Load responses for all specimens: (**a**) *F-*CMOD responses; and (**b**) *F*-Δ responses.

Figure 6a,b show the *F*-CMOD and *F*-Δ responses, respectively, for all the specimens. From Figure 6a, it can be observed that all the specimens have similar responses up to the peak load, and the post-peak responses of PN_B_X specimens start to deviate from the responses of CN_X and PN_X specimens (without the bar) as the GFRP bar engagement increases. The reason behind all the specimens having similar initial trend is because the effect of the GFRP bar is minimal before the post-peak ascending part of the responses. This phenomenon is further explained later in this Section.

### 4.2. Responses from the Free End of the GFRP Bar

**Figure 7:**
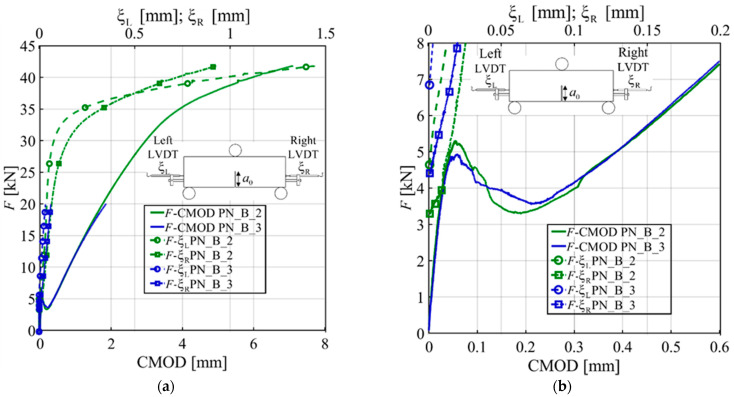
Plots of free end movement of GFRP bars: (**a**) *F* is plotted against CMOD and ξ_R_ and ξ_L_ for PN_B_2 and PN_B_3; (**b**) A call out for the first segment of Figure 7a.

Specimens PN_B_2 and PN_B_3 were equipped with two LVDTs attached to the protruding part of the GFRP bar beyond the concrete beam end cross-sections to measure the free end slip of the bar with respect to concrete. A similar measurement was achieved in the pull-out tests recently published by the authors [5]. Figure 7a shows the plot of *F* versus CMOD and *F* versus the left and right free end slips of the bar ξ_R_ and ξ_L_, respectively. ξ_R_ and ξ_L_ are plotted separately to visualize the different behavior of each portion of the bar separated by the bond breaker at midspan.

In Figure 7a, the response of PN_B_2 shows that up to 37 kN ξ_R_ > ξ_L_ for the same load level. However, when *F* > 33 kN, the *F*-ξ_R_ and *F*-ξ_L_ show a different trend. The change in the response seems to be associated with the end of the almost-linear part of the ascending part of the *F*-CMOD response described above. Eventually, at the end of the test, the left free end of the bar exhibited larger slips compared to the right end. Figure 7b is a call out of Figure 7a. It shows that the free ends of the bar started to slip at different values of the load, which ranged between 3 kN and 7 kN. This range is consistent with the corresponding range in the pull-out tests of the same bar with similar bonded length (240 mm) [5].

**Figure 8:**
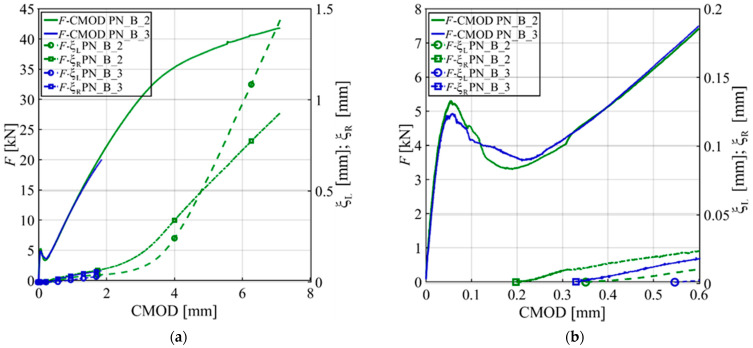
Plots of free end movement of GFRP bars: (**a**) CMOD is plotted against *F* and ξ_R_ and ξ_R_ for PN_B_2 and PN_B_3; (**b**) A call out for the first segment of Figure 8a.

In Figure 8, the same responses of Figure 7 are used, but *F*, ξ_R_, and ξ_L_ are plotted against the CMOD. Figure 8a shows that the ξ_R_-CMOD and ξ_L_-CMOD responses have an initial linear portion followed by a nonlinear response that match with the *F-*CMOD linear and nonlinear responses in the post-peak ascending part. Figure 8b is a call out of Figure 8a. Figure 8b shows that the slip of the free ends of the bar starts at the beginning of the post-peak ascending part of the *F-*CMOD response. This observation supports the previous statement regarding the bar engagement in the post-peak descending part. It is reasonable to assume that the contribution of the bar to the load bearing capacity of the notched beam before the peak is limited.

### 4.3. F-Δ Responses from Digital Image Analysis

Digital image correlation (DIC) was used on five out of nine notched beam specimens to obtain the displacement field and, eventually, the strain field on one of the longitudinal side faces of the specimens [33]. To perform the correlation, a subset size of 41 pixels (approximately 7.5 mm, which is smaller than the maximum aggregate size equal to 12 mm) and a step of 10 pixels (approximately 2 mm) were chosen. For the PN_2 specimen (the only specimen with DIC in the PN_X series), DIC analysis was not successful due to a synchronization problem between the DIC images and the load acquisition system; thus, this specimen is disregarded in the remainder of the paper.

**Figure 9:**
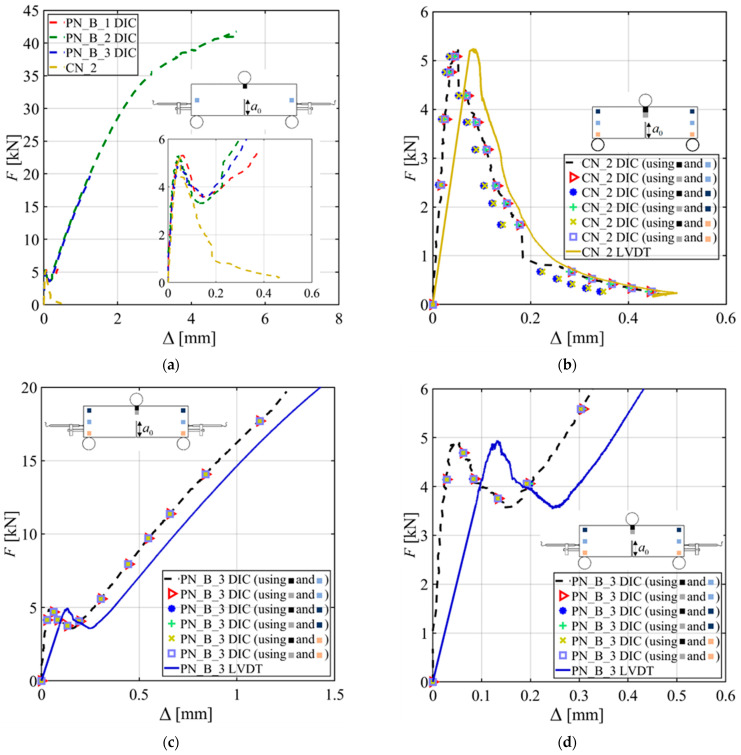
Load responses from DIC analysis: (**a**) *F*-Δ for all the specimens equipped with DIC; (**b**) Comparison of *F*-Δ responses from DIC analysis and LVDTs for CN_2; (**c**) Comparison of *F*-Δ responses from DIC analysis and LVDTs for PN_B_3; (**d**) Call out for the first portion of Figure 9c.

*F*-Δ responses were obtained from the DIC analysis by using the displacement field. The load point displacement (Δ) was computed by subtracting the average of the vertical displacements at the supports from the vertical displacement at the point of loading [34]. The vertical displacement at each support was computed by averaging the vertical displacements within an 8 mm square area centered at mid-height of the support cross-section. The vertical displacement of the loading point was also computed by averaging the vertical displacements within an 8 mm square area just underneath the Z-shaped plate location. It should be mentioned that for all the specimens, the vertical displacements at the supports were consistent with each other. Figure 9a shows the *F*-Δ responses from DIC analysis for all the specimens. In the call out of Figure 9a, the initial linear part of the response for all specimens appears to be consistent. The *F*-Δ responses from LVDTs, as pointed out earlier when referring to Figure 4c,d, were missing this consistency in the initial slope. Furthermore, in Figure 9b–d (which is a call out of Figure 9c), the *F*-Δ responses from DIC analyses are compared with the *F*-Δ responses from LVDTs to show the difference between the responses for specimens CN_2 and PN_B_3. These plots show that the LVDT displacements were larger than those from DIC due to the previously mentioned reasons. Researchers have argued that the computation of the vertical displacement considering the location just underneath the loading point can be misleading due to stress concentration [34]. Similarly, there might be some stress concentration at support locations as well, where the squares were taken to compute the vertical displacement at support locations. To explore this phenomenon, the square used to compute the vertical displacement at the midspan was moved downward. Additionally, the squares used to compute the vertical displacement at the supports were moved both toward the bottom and top edges of the specimen. A combination of these square locations at the midspan and at the supports was considered, and the *F*-Δ responses are reconstructed for each combination of squares for specimens CN_2 and PN_B_3 in Figure 9b–d. From these plots, it can be observed that the location of the squares at the midspan and at the supports has little to no effect on the *F*-Δ responses.

## 5. Failure Modes

For all the specimens, micro-damage occurred at the tip of the notch before attaining the peak load (*F_max_*). However, the crack started to propagate in the descending part of the load response. For the CN_X and PN_X specimens, crack propagation was associated with a long tail in the post-peak descending part of the load response. The test was stopped when the load was approximately 4% of *F_max_*. For the PN_B_X specimens, the larger values of the CMOD in the post-peak ascending part of the responses (when compared to CN_X and PN_X specimens) were due to the bridging action of the GFRP bar and the possibility of carrying tensile stresses by the bar itself.

**Figure 10:**
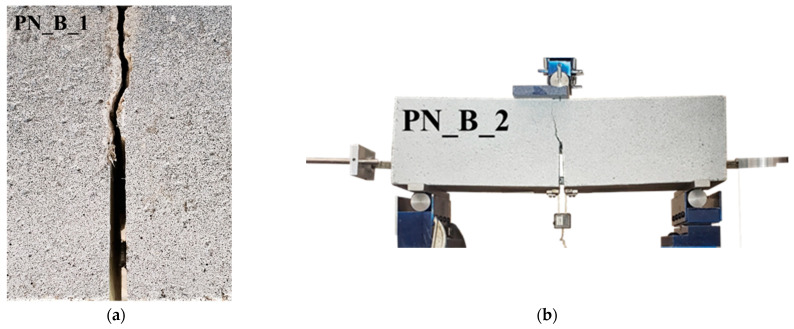

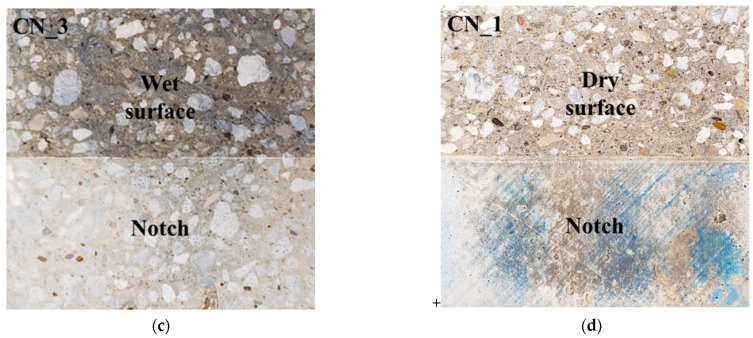
(**a**) Tortuous crack path of PN_B_1; (**b**) Picture of the PN_B_2 specimen just before the test was stopped; (**c**) Wet fracture surface of CN_3; (**d**) Dry fracture surface of CN_1.

For the PN_B_X specimens, the tests were continued until the supporting cylinders were deemed to be in a safe position after rolling. The crack paths were straight (i.e., the crack propagated from the tip of the notch along the ligament line) for all the CN_X and PN_X specimens. However, the crack paths were slightly tortuous for the PN_B_X specimens [7]. For PN_B_1 and PN_B_4, an initial deviation with different angles from the straight pattern was observed near the notch tip. As the crack propagated further, its path gradually straightened up to become closer to the ideal line of the ligament. It is possible that the crack paths for the PN_B_X specimens were deviating from the ideal path due to the presence of the GFRP bar and the difference in the slips between the GFRP bar and concrete in the portions on the left and right of the notch as mentioned earlier in Section 3. For specimens PN_B_2 and PN_B_3, the crack path continued to deviate from the ideal straight path until it reached the top of the beam. In Figure 10a, the crack path of specimen PN_B_1 is shown as a representative case. Figure 10b shows the final configuration and fractured surface of specimen PN_B_2; the test was stopped after this point.

Most of the fracture surfaces of the CN_X and PN_X specimens were still wet after the test; however, the fracture surfaces of specimens CN_1 and PN_1 were slightly drier compared to the other specimens, although not fully dry. In Figure 10c,d, representative cases of the fracture surfaces are shown. It should be mentioned that the peak load of specimens CN_1 and PN_1 was slightly larger than the peak load of the other specimens within their respective series.

The effect of the moisture content on the fracture behavior of concrete is discussed in [32]. The fracture surfaces of the PN_B_X specimens were not accessible to examine, as these specimens were not broken into two halves after the test.

## 6. Discussion

The results presented in this paper and the following analysis refer to a specific type of bar. Further research is needed to investigate the bond behavior of FRP bars with different mechanical and physical properties (for example, different surface treatment and type of resin and fiber used).

### 6.1. Fracture Energy

For the CN_X and PN_X specimens, the fracture energy (*G_F_*) was computed using the area under the *F*-Δ curve [19,22,35]. Since no weight compensation technique was employed during the tests, the effect of the self-weight (*F*_0_) of the specimens was taken into account [36]. For the sake of brevity, the procedure to account for the self-weight is not explained here in full length, and the reader can refer to a previous work [32]. The fracture energy was estimated as [19]

(2) $$G_{F}=\frac{W_{F}}{BD(1-\alpha_{0})}$$

where *W_F_* is the area under the *F-*Δ curve after adjusting for the self-weight, and $\alpha_{0}$ is equal to *a*_0_/*D*. To compute *G_F_* correctly, the end of the tail (to zero load) is required. However, practically, it is not possible to run the test until the load is zero, since the notched beam would break down before it reaches zero load. To tackle this problem, the end tail portion of the response was extrapolated by using the function $Y=ae^{-bX}$ (which is integrable up to ∞). *Y* is the load, *X* is the load point displacement, and *a* and *b* are constants that are calibrated by fitting the existing experimental part of the tail response. The fracture energy (*G_F_*) for the series CN_X and PN_X is reported in Table 3 along with its average (${\bar{G}}_{F}$) and coefficient of variation. The average fracture energies for the CN_X and PN_X specimens are consistent. This indicates that there was no significant impact on the fracture behavior whether the initial notch was cast or cut with a blade. The consistency in the peak load between the CN_X and PN_X specimens (CoV of peak loads of these two series of specimens combined is 0.054) is another indication that the process to create the notch does not affect the fracture response.

### 6.2. Elastic Modulus from CMOD

The elastic modulus was computed from the initial linear elastic part (30% to 60% of the peak load) of the *F*-CMOD response using the following formula [37]:

(3) $$\mathrm{CMOD}=\frac{6FS}{E_{\mathrm{CMOD}}BD^{2}}a_{0}V_{\beta }(\alpha_{0})$$

where *V*_β_(α_0_) is a dimensionless function that depends on α_0_ and

(4) $$V_{\beta }(\alpha_{0})=0.8+1.7\alpha_{0}+2.4\alpha_{0}^{2}+\frac{0.66}{{\left(1-\alpha_{0}\right)}^{2}}+\frac{4}{\beta }(-0.04-0.58\alpha_{0}+1.47\alpha_{0}^{2}-2.04\alpha_{0}^{3})$$

where β = *S*/*D*. For all specimens in this paper, β = 3. The elastic modulus (*E*_CMOD_) was computed for all the specimens and reported in Table 3 along with its average and CoV. The consistency of *E*_CMOD_ values between the CN_X and PN_X specimens again proves that casting the notch rather than saw cutting it did not have any impact on the fracture properties of the specimens. Moreover, the elastic modulus values of the PN_B_X series specimens are also consistent with the CN_X and PN_X specimens, which, again, indicates that in the first linear part of the response, there is a limited effect of the GFRP bar.

### 6.3. Strain and Displacement Profiles Using Digital Image Analysis

In this Section, the horizontal strain ε*_xx_* and displacement Δ*u_x_* profiles along the notch ligament are discussed. These profiles were obtained from the DIC analysis. The Cartesian coordinate system shown in Figure 3d was used in this Section. The ε*_xx_* profile was constructed by averaging the values of the horizontal strain component within square areas along the ligament of the notch. The size of the squares was 5 mm by 5 mm. The average horizontal strain within the square was computed and plotted against the normalized values of *y*, i.e., *y*/(*D* − *a*_0_), corresponding to the centroid of the square.

**Figure 11:**
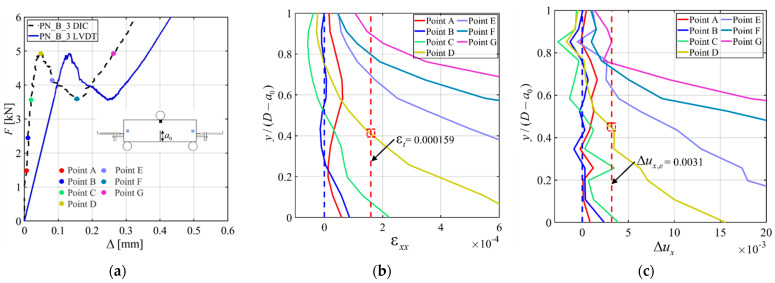
ε*_t_* and Δ*u_x_* profiles for PN_B_3: (**a**) Loading points shown in colored circle for ε*_xx_* and Δ*u_x_*; (**b**) ε*_xx_* profile; and (**c**) Δ*u_x_* profile.

Figure 11a shows the *F*-Δ responses from DIC and LVDT for specimen PN_B_3. Figure 11b shows the ε*_xx_* profile for the same specimen. The different colored curves correspond to different loading points shown with the same-color circled markers in the *F*-Δ response from DIC reported in Figure 11a. In the strain profile plot of Figure 11b, the blue dashed vertical line represents the ligament of the notch, and the red dashed vertical line is the strain (ε*_t_*) value corresponding to the tensile strength of concrete. ε*_t_* was computed as

(5) $$\varepsilon_{t}=\frac{{f^{\prime }}_{t}}{{E^{\prime }}_{\mathrm{CMOD}}}$$

where ${E^{\prime }}_{\mathrm{CMOD}}$ is the average of *E*_CMOD_ for all specimens reported in Table 3. Using Equation (5), the magnitude of ε*_t_* was equal to 0.000159. In Figure 11b, the strain curve corresponding to the peak load (Point D) intersects with the ε*_t_* vertical dashed line at *y*/(*D* − *a*_0_) = 0.42, which is marked with a red square marker. This normalized *y* value indicates the normalized length of the fracture process zone (FPZ) at the peak load [38]. Similarly, for the other strain profiles, the intersection with the ε*_t_* line indicates the length of the FPZ for the corresponding load points. Figure 11b shows that, at the peak load, the FPZ reaches almost half of the ligament length. It also shows that the FPZ starts to form just after the end of the initial linear part of the response. The intersection of the blue dashed line with the ε*_xx_* profile provides the location of the neutral axis for the corresponding load point. It should be mentioned that strain profiles corresponding to points of the load response in the post-peak ascending part were deemed unnecessary, as the neutral axis was basically almost coincident with the outermost fibers of the beam. As the neutral axis reached the outermost fibers on top, averaging the strain over the square provided values of the strain component ε*_xx_* that were not representative of the actual strain near the top fibers because of the complexity of the stress state. Figure 11c shows the Δ*u_x_* profiles for specimen PN_B_3. Δ*u_x_* was computed by taking two squares on each side of the notch tip. The side of the squares was 5 mm. The squares were placed 10 mm apart on center. The averages of the horizontal displacements within the squares were then computed and subtracted from each other to obtain Δ*u_x_*. This process was performed along the whole ligament and then plotted against the normalized value of *y*. The different colored curves in Figure 11c again represent the different load points marked in Figure 11a. The blue dashed line is again the ligament, and the red dotted line is the maximum elastic elongation Δ*u_x,e_*.

The maximum elastic elongation was computed as

(6) $$\Delta u_{x,e}=\varepsilon_{t}\chi$$

where χ is the center-to-center distance (i.e., 10 mm) of the two squares considered to compute Δ*u_x_*. The value of the Δ*u_x,e_* was equal to 0.0031 mm. In Figure 11c, the curve corresponding to the peak load (Point D) intersects Δ*u_x,e_* at *y*/(*D* − *a*_0_) = 0.43, which, again, indicates the size of the FPZ for that load. It is observed that the sizes of the FPZ at the peak load determined from the ε*_xx_* and Δ*u_x_* profiles are consistent. This consistency between these two profiles can also be found for the other load points as well. At the beginning of the post-peak ascending part of the response of Figure 11a (Point F), where it is assumed that the bar engagement increased, the FPZ almost exploit 80% of the ligament length (Figure 11b,c). This indicates that the crack propagated significantly at that load point.

**Figure 12:**
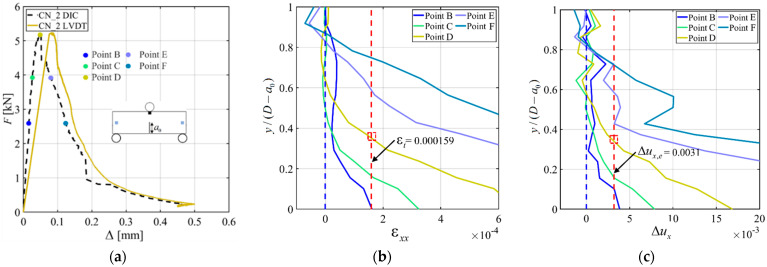
ε*_xx_* and Δ*u_x_* profiles for CN_2: (**a**) Loading points shown in colored circle for ε*_xx_* and Δ*u_x_*; (**b**) ε*_xx_* profile; and (**c**) Δ*u_x_* profile.

**Figure 13:**
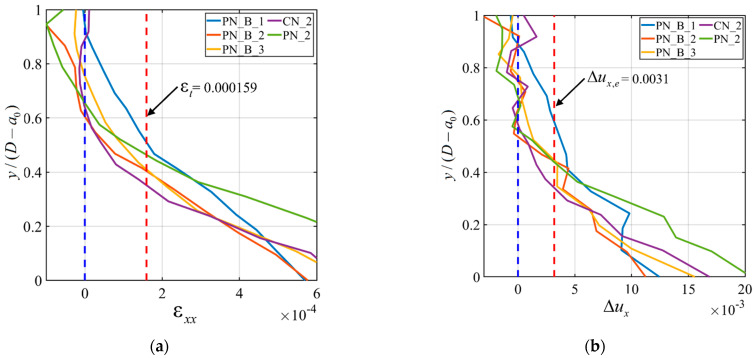
(**a**) ε*_xx_* profile for all specimens equipped with DIC considering only peak loads; and (**b**) Δ*u_x_* profile for all specimens equipped with DIC considering only peak loads.

The ε*_xx_* and Δ*u_x_* profiles were constructed for specimen CN_2 and are reported along with the *F*-Δ response from DIC in Figure 12a–c. As for specimen PN_B_3, the length of the FPZ at the peak load (Point C) was determined from the ε*_xx_* profile, and it was *y*/(*D* − *a*_0_) = 0.36 for CN_2. A similar size of the FPZ at the peak load was obtained from the Δ*u_x_* profile. The size of the FPZ at the peak load for specimens CN_2 is slightly smaller than that of specimen PN_B_3. To further study the effect of the GFRP bar on the size of the FPZ at the peak, the ε*_xx_* and Δ*u_x_* profiles corresponding to the peak load for all specimens (with DIC analysis) are plotted together in Figure 13a,b, respectively. Figure 13 shows that there is no clear trend that could be linked to the influence of the GFRP bar on the FPZ size at the peak. This observation confirms that the bar engagement and, therefore, its effect on the load response increased after the peak load.

### 6.4. Force in the GFRP Bar and Comparison with the Pull-Out Test

**Figure 14:**
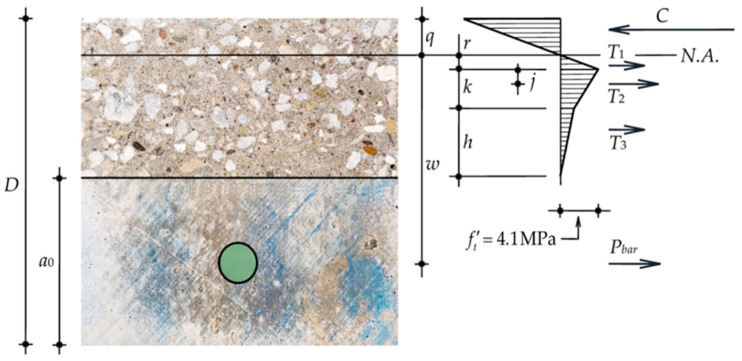
A typical cross-section analysis for PN_B_X series.

The force in the GFRP bar (*P_bar_*) in the PN_B_X specimens was computed using the moment equilibrium [39] and DIC analysis. The cross-section stress profile at the midspan of the PN_B_X specimen is shown in Figure 14. The compressive force and the tensile force in the uncracked concrete block are denoted as *C* and *T*_1_, respectively. The cohesive zone was defined using Petersson’s bilinear softening curve [40]. The tensile forces in the cohesive zone are denoted as *T*_2_ and *T*_3_.

The ε*_xx_* profile from DIC analysis (Figure 11b) was used to associate the location of the neutral axis (N.A.) and the length of the FPZ to each value of the applied load. The N.A. was found from the intersection of the ε*_xx_* curve and the ligament line.

The beginning of the cohesive zone was found as the intersection of the ε*_xx_* curve and the ε*_t_* vertical line. The Δ*u_x_* profile was used to determine the crack openings within the FPZ and associate the stress provided by Petersson’s bilinear softening curve [40]. Δ*u_x_* was assumed equal to the crack opening. The moment equilibrium was then applied to solve for *P_bar_*:

(7) $$P_{bar}=\frac{\frac{3FD}{4}-\frac{2T_{1}}{3}(q+r)-T_{2}(\frac{2q}{3}+r+j)-T_{3}(\frac{2q}{3}+r+k+\frac{h}{3})}{w+\frac{2q}{3}}$$

where *q* and *r* are the lengths of the compressive and tensile region, respectively, for the uncracked concrete block, and *w* is the distance from the N.A. to the centroid of the GFRP bar. *k* and *h* are the lengths of the tensile regions corresponding to the forces *T*_2_ and *T*_3_, and *j* is the distance of *T*_2_ to the beginning of the FPZ. The slip of the GFRP bar (*g*) at the beginning of the bonded area was determined from DIC analysis. The difference of the horizontal displacements between the two points on each side of the notch corresponding to the beginning of the bonded areas was named Δ*u_l_* and was obtained from DIC analysis. Two squares (10 mm side) on each side of the notch where the bond breaker ended were used to average the horizontal displacements. The difference of the averages of the displacements corresponded to Δ*u_l_*. The slip *g* was then obtained by subtracting the elastic deformation of the bar from Δ*u_l_* and assuming an equal slip on each side of the notch:

(8) $$g=\frac{\Delta u_{l}-\frac{P_{bar}l}{A_{bar}E_{bar}}}{2}$$

As noted above, *l* is the length of the unbonded length due to the presence of the bond breakers.

**Figure 15:**
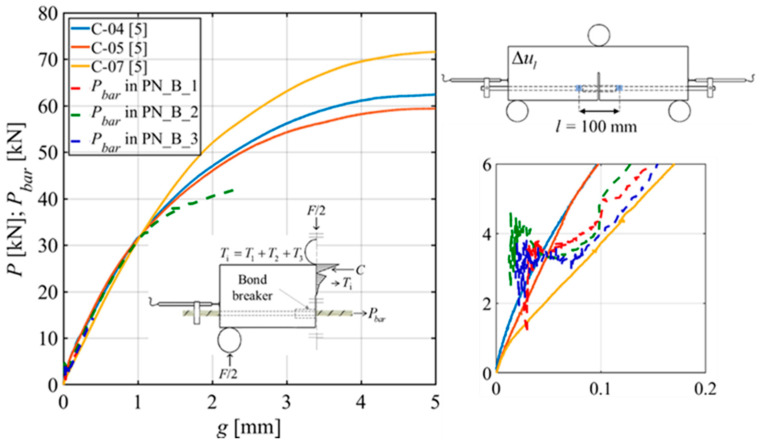
Force in the GFRP bar versus the slip obtained in PN_B_X specimens and in pull-out tests with a 200 mm bonded length.

**Figure 16:**
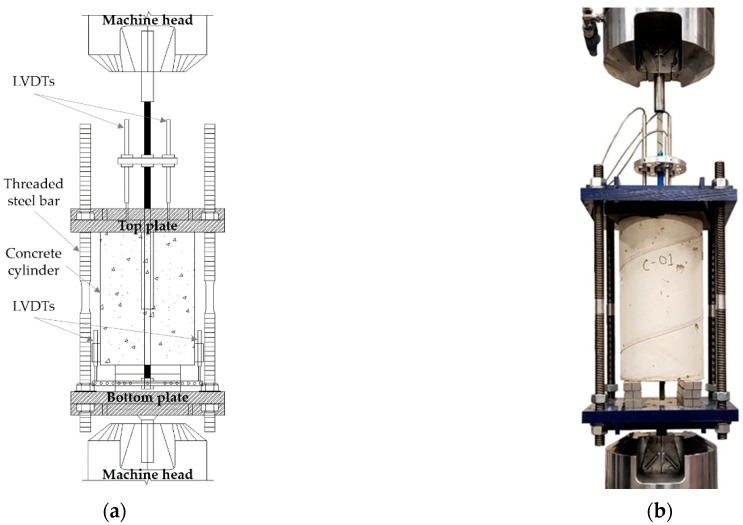
(**a**) Schematic of the pull-out test set-up; (**b**) Photo of the test [5].

Figure 15 shows the plot of the force in the GFRP bar (*P_bar_*) versus the slip *g* for the three PN_B_X specimens for which DIC analysis was available. Figure 15 includes the applied load *P* versus the loaded end slip *g* from three pull-out tests of the same GFRP bar embedded in concrete cylinders. These plots refer to an experimental campaign conducted by the authors to study the effect of the bonded length in pull-out tests of GFRP bars [5]. The diameter and the length of the concrete cylinders used for the pull-out tests were 200 mm and 380 mm, respectively. The GFRP bar was placed at the center of the cylinder. A bond breaker was created using the same PVC pipe used for the notched beam bond breakers. The push–pull test set-up resembled that of ASTM D7913 [41]. A schematic of the pull-out test set-up is shown in Figure 16a together with a photo in Figure 16b.

Threaded hollow cylinders were glued at the top end of the bar to improve the gripping of the GFRP bar by the semi-moon jaws of the servo-hydraulic machine. The concrete cylinder reacted against a 25 mm thick steel plate on the top named the *top plate*. The *top plate* had a hole at the center to pass the GFRP bar through, and it was connected to another plate on the bottom of the cylinder called the *bottom plate*. The *top plate* was connected with the *bottom plate* by 22 mm diameter threaded steel bars featuring frictionless bearings to reduce torsion. This bottom plate was connected to the jaws of the servo-hydraulic machine by means of a square steel plate and a stud. Three LVDTs were attached to the top part of the GFRP bar (120° apart) at a distance of 130 mm from the top surface of the concrete cylinder. The average of these three LVDTs was used to control the test. The slip *g* for the pull-out tests was computed by subtracting the GFRP bar deformation (between the LVDTs and the beginning of the bonded area) from the average of the LVDT readings.

The bonded length of the pull-out tests was 240 mm. The bonded length on each side of the notch in the beams was 225 mm. Even though the bonded length in the pull-out test was slightly larger than the bonded length of the GFRP bar in each half of the notched beams, the comparison of the pull-out test responses with the *P_bar_* versus *g* plots shows that the responses are in good agreement with each other. Interestingly, the *P_bar_* versus *g* response of specimen PN_B_2 indicates that the load in the notched beam increases at a different rate with respect to *g* when compared to the force in the bar in pull-out tests, which suggests that the pull-out tests could be considered an upper bound in terms of the bond behavior of GFRP bars in structural elements. The reason behind this observation could be the effect of bending that is present in the TPB tests of notched beams, which could imply a non-uniform distribution of the interfacial shear stresses along the perimeter of the bar.

### 6.5. Contour Plots from Digital Image Analysis

**Figure 17:**
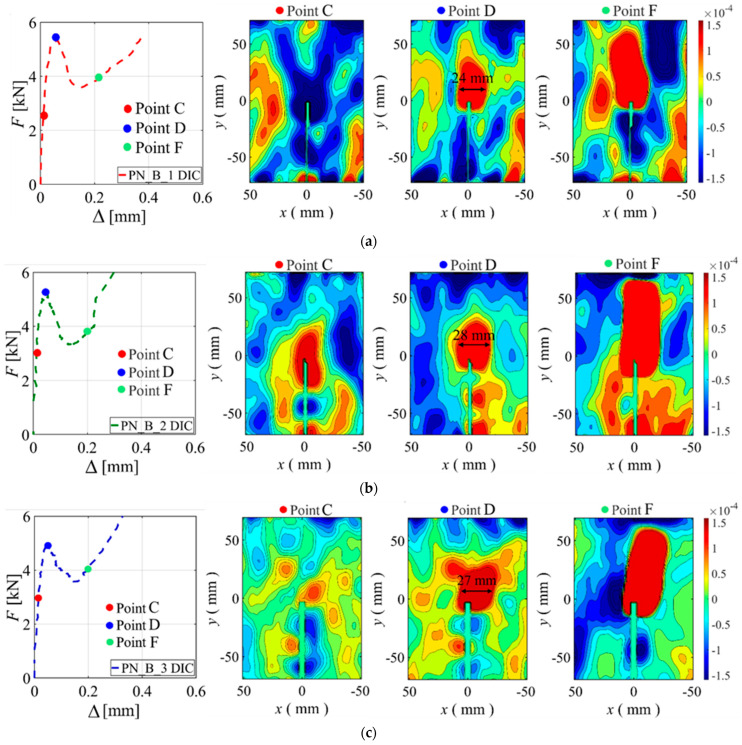

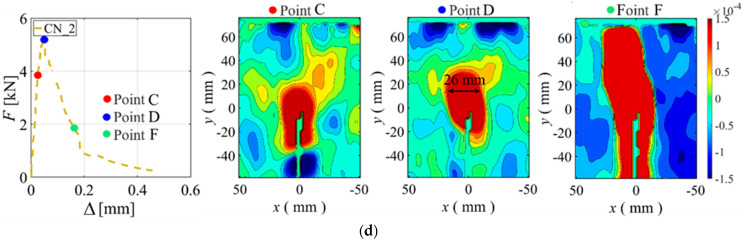
Contour plots of ε*_xx_* considering three different loading points: (**a**) PN_B_1; (**b**) PN_B_2; (**c**) PN_B_3; and (**d**) CN_2.

The contour plots of the horizontal strain component (ε*_xx_*) are shown in Figure 17a–d. The reference system for these plots is the Cartesian coordinate system shown in Figure 3d. The area of interest for the contour plots is a rectangle of 50 mm width and 150 mm height centered at the tip of the notch. The choice of the area of interest was to investigate the width of the FPZ at different load points. For the PN_B_X series, three contour plots are shown corresponding to three points of the load response. These points correspond to 50% of the peak load in the linear elastic part (Point C), the peak load (Point D), and the beginning of the post-peak ascending response (Point F). The load points are shown with colored circled markers in the corresponding *F*-Δ plots in Figure 17a–c. The width of the FPZ measured at the peak loads for specimens PN_B_1, PN_B_2, and PN_B_3 is 24 mm, 28 mm, and 27 mm, respectively. In addition to the contour plots of the PN_B_X series, Figure 17d shows the contour plots of specimen CN_2. Three points of the load response of CN_2 were considered: (1) 75% of the peak load in the linear elastic part; (2) the peak load; and (3) the load corresponding to the same CMOD value as the PN_B_X specimen’s Point F. At the peak load, the width of the FPZ for CN_2 is 26 mm. Based on the presented data, the size of the FPZ does not seem to be affected by the GFRP reinforcement in the beams.

## 7. Conclusions

Three-point bending tests of concrete notched beams with and without a GFRP bar as internal reinforcement were carried out using digital image correlation analysis for some of the specimens. The fracture tests were used to determine the fracture behavior of the beams and the effect of the internal reinforcement on the crack propagation, crack mouth opening displacement (CMOD), and crack pattern. In addition, the load in the bar and the slip at the beginning of the bonded area were determined using DIC and cross-sectional moment equilibrium and compared with the results of the pull-out tests. The notch was cast for specimens with the GFRP bar and either cast or cut for specimens without the bar. The following conclusions can be outlined:

- For notched beams without the GFRP reinforcement, the load responses and peak loads of specimens with cast or saw-cut notch were consistent and indicated that both solutions are acceptable to determine the fracture response of concrete.
- Notched beams reinforced with a GFRP bar exhibited an initial response almost identical to that of specimens without the GFRP bar until the first peak. After the peak, beams without the bar had a long-tail descending part until nominally zero load was reached. On the other hand, the response of the beams with the GFRP bar deviated from that of the beams without the bar in the descending part and started to increase again, which indicated the increasing engagement of the bar.
- The computation of the fracture energy *G_F_* of concrete (using the beams without the bar) was not affected by whether the notch was cast or saw cut.
- The computation of the elastic modulus from the initial linear branch of the load versus CMOD response indicated that the bar had a limited effect on the load response prior to the attainment of the first peak load.
- The size of the fracture process zone (FPZ) did not seem to be affected by the presence of the GFRP bar.
- The crack path appeared to be more tortuous for the beams reinforced with the GFRP bar.
- The force in the bar versus the slip at the beginning of the bonded area in notched beams was compared with the load versus loaded end slip of the pull-out tests. It was concluded that, in terms of the bond behavior, pull-out tests could be assumed as an upper bound with respect to the bond phenomenon in structural elements where bending is present.

## Figures and Tables

**Table 1:** Results of the tensile tests of GFRP bars.

GFRP Bar	Ultimate Load [kN]	Tensile Strength [MPa]	Average Tensile Strength [MPa] (CoV)
1	129.40	1019	1025 (0.004)
2	130.05	1024	
3	131.81	1030	

**Table 2:** Specimen dimensions and peak loads.

Specimen	*D* (CoV) [mm]	*B* (CoV) [mm]	*L* (CoV) [mm]	*S* [mm]	Weight [N]	*a*_0_ (CoV) [mm]	*F_max_* [kN]	${\bar{F}}_{max}$ (CoV) [kN]
PN_B_1	152.4 (0.012)	557.2 (0.001)	155.6 (0.011)	457.2	296.3	76.2 (n/a)	5.32	5.22 (0.036)
PN_B_2	155.6 (0.016)	557.2 (0.004)	152.4 (0.000)	457.2	295.3	76.2 (n/a)	5.31	
PN_B_3	150.8 (0.011)	557.2 (0.002)	155.6 (0.012)	457.2	289.4	76.2 (n/a)	4.89	
PN_B_4	152.4 (0.006)	558.8 (0.002)	152.4 (0.000)	457.2	293.3	76.2 (n/a)	5.36	
CN_1	157.2 (0.016)	562 (0.002)	157.2 (0.011)	457.2	307.1	79.4 (0.043)	5.65	5.38 (0.035)
CN_2	155.6 (0.006)	562 (0.001)	154.0 (0.006)	457.2	305.1	79.4 (0.021)	5.24	
CN_3	157.2 (0.006)	562 (0.002)	155.6 (0.006)	457.2	305.1	77.8 (0.021)	5.26	
PN_1	158.8 (0.012)	560.4 (0.000)	155.6 (0.006)	457.2	310.9	76.7 (0.012)	5.58	5.21 (0.071)
PN_2	157.2 (0.012)	560.4 (0.002)	155.6 (0.006)	457.2	305.1	75.7 (0.012)	4.84	

**Table 3:** Fracture energy (*G_F_*) and elastic modulus (*E*_CMOD_) from CMOD.

Specimen	$G_{F}$ [N/m]	${\bar{G}}_{F}$ [N/m] (CoV)	$E_{\mathrm{CMOD}}$ [GPa]	${\bar{E}}_{\mathrm{CMOD}}$ [GPa] (CoV)
PN_B_1	-	-	30.3	25.9 (0.108)
PN_B_2	-		25.9	
PN_B_3	-		25.0	
PN_B_4	-		22.5	
CN_1	74.3	81.3 (0.111)	26.8	25.5 (0.043)
CN_2	75.2		25.5	
CN_3	93.8		24.1	
PN_1	90.2	86.2 (0.045)	28.8	27.2 (0.058)
PN_2	82.3		25.6	

## Data Availability

The data presented in this study are available upon request from the corresponding author.
